# Assessing the environmental impact of post-revolution reforms in Tunisia: a synthetic control approach

**DOI:** 10.1186/s13021-026-00417-5

**Published:** 2026-02-24

**Authors:** Mehdi Ben Jebli, Adel Benhamed

**Affiliations:** 1 FSJEG Jendouba, University of Jendouba, Jendouba, Tunisia; 2 QuAnLab LR24ES21. ESCT, Campus University of Manouba, 2010 Manouba, Tunisia; 3 School of Business, King Faisal University, 31982 AlAhsa, Saudi Arabia

**Keywords:** CO_2_ emissions, Energy transition, Tunisia, Synthetic control method

## Abstract

Understanding how major institutional and economic reforms influence environmental outcomes is essential for countries undergoing political transitions. This study examines how Tunisia’s CO₂ emissions trajectory evolved during the post-revolution institutional transition relative to a synthetic counterfactual constructed using the Synthetic Control Method (SCM). Rather than identifying the causal effectiveness of specific reforms, the analysis assesses whether Tunisia’s emissions trajectory diverged from that of a comparable synthetic unit following the reforms implemented after 2014 within a robust counterfactual framework. A synthetic version of Tunisia is constructed using a weighted combination of comparable North African countries, Algeria, Egypt, Libya, Morocco, and Sudan, based on pre-intervention data from 2000 to 2013 for key predictors, including GDP, renewable energy consumption (REC), non-renewable energy consumption (NREC), and foreign direct investment (FDI) inflows. The SCM results indicate that, following the post-2014 institutional transition, Tunisia’s CO₂ emissions trajectory diverged from its synthetic counterpart, with observed emissions rising more rapidly over the study period. While the estimated gap reaches approximately 58.3% by the end of the sample, placebo tests and RMSPE ratios indicate that this divergence is only weakly distinguishable from donor-country placebos, underscoring the need for cautious interpretation. These findings indicate that the post-revolution transition did not coincide with an immediate or statistically robust reduction in CO₂ emissions relative to the counterfactual, rather than providing definitive evidence of reform success or failure. From a policy perspective, the results highlight the importance of aligning institutional and economic reforms with clearly operationalized climate and energy policies. In particular, accelerating renewable energy deployment, improving energy efficiency, phasing down fossil-fuel subsidies, and strengthening regulatory and governance frameworks are essential to ensure that future reform efforts translate into meaningful environmental improvements. The findings provide critical insights for Tunisia and other North African countries seeking to balance economic development with environmental sustainability during periods of institutional transition.

## Introduction

The relationship between economic progress and environmental protection is a critical challenge worldwide, particularly for developing countries like Tunisia, which has undergone major political and economic transformations since 2011. While making strides toward democratic governance and economic improvement, Tunisia continues to face environmental challenges, notably rising carbon dioxide (CO₂) emissions [22]. This introduction examines Tunisia’s environmental state by focusing on CO₂ emission trends, underlying causes, governmental efforts to address environmental issues, and the broader transition toward cleaner energy sources.

Tunisia’s environmental challenges are shaped by several factors, including increased industrial activity, urban expansion, and evolving energy consumption patterns. A primary driver of rising CO₂ emissions is the country’s heavy reliance on fossil fuels for electricity generation, transportation, and industrial uses [18], exacerbated by growing energy demand from economic and population growth. Recent data from the Agence Nationale pour la Maîtrise de l’Énergie [6] indicate that energy consumption continues to rise, outpacing gains in efficiency and underscoring the urgent need for cleaner energy sources. Beyond energy production, other sectors significantly contribute to emissions: the transportation sector, affected by increasing vehicle ownership and freight transport, remains a major source [41], while industrial activities such as cement and chemical production add further emissions [10]. Land use changes, including deforestation and agricultural expansion, also elevate greenhouse gas emissions by diminishing the natural absorption capacity of the environment.

To address environmental challenges, the Tunisian government has implemented various policies to reduce CO₂ emissions and promote sustainability. A landmark step was the 2014 constitution, which recognized environmental protection as a fundamental right and paved the way for institutional reforms [46]. Subsequently, the government launched initiatives to diversify energy sources, attract foreign investment in renewables, and foster sustainable economic growth. Financial support has been key to these efforts, facilitating the adoption of renewable technologies through private sector investment incentives, loans to green businesses, and innovation funds [15, 37]. Policies promoting renewables, especially solar and wind, are reinforced by regulatory incentives [24]. The Tunisian Solar Plan aims to increase renewables’ share in electricity to 30% by 2030 [38], alongside efforts to improve energy efficiency in buildings and transport, encourage sustainable agriculture, and strengthen environmental regulations.

A central part of Tunisia’s strategy to combat climate change is its commitment to transitioning away from fossil fuels and towards renewable energy sources. The government has set ambitious targets to increase the share of renewables in the energy mix and developed a comprehensive national plan focusing on promoting renewable technologies, enhancing energy efficiency, and reducing greenhouse gas emissions [45]. This energy transition aligns with broader economic goals emphasizing green growth and sustainable development. To support these objectives, Tunisia has implemented policies to attract foreign investment in renewable projects, encourage environmentally friendly technologies, and foster job creation in green industries.

In this study, the ‘post-2014 institutional and economic reforms’ that constitute the Synthetic Control Method (SCM) treatment are explicitly defined as the major governance, regulatory, and energy-sector interventions implemented after the adoption of the 2014 Constitution. These include: (i) governance reforms such as the constitutional reorganization of public institutions, the launch of the decentralization process (2015–2016), and the adoption of new transparency and accountability frameworks; (ii) energy-sector reforms including the gradual reduction of fossil-fuel subsidies (2014–2017), the introduction of the Renewable Energy Law (Law n°2015-12) permitting independent power producers, and the establishment of fiscal incentives to attract private investment in solar and wind projects; and (iii) regulatory reforms aimed at improving the investment climate through updated competition laws, simplified licensing procedures, and improved public financial management. Together, these coordinated reforms form a clearly identifiable intervention that began in 2014 and intensified between 2015 and 2017, making this period the treatment window used in the SCM.

To conceptualize the link between institutional reforms and environmental performance, this study draws on several established theoretical frameworks. Ecological Modernization Theory suggests that structural reforms, improved regulatory capacity, and institutional innovation can enable countries to decouple economic growth from environmental degradation through cleaner technologies and more efficient resource use. Complementing this view, Institutional Environmentalism emphasizes the role of governance quality, policy coherence, and administrative capacity in shaping environmental outcomes, arguing that institutional reforms can either strengthen or weaken the enforcement of environmental regulations. Additionally, although this study does not estimate an Environmental Kuznets Curve (EKC) structure, the EKC framework offers useful intuition regarding how economic and institutional development may alter emissions trajectories over time. Together, these perspectives provide a conceptual foundation for understanding how Tunisia’s post-2014 reforms may have influenced CO₂ emissions through changes in regulatory enforcement, investment behavior, and the alignment of economic activity with environmental objectives.

More specifically, this study focuses on the set of institutional, governance, and energy-related reforms implemented in Tunisia after the 2014 Constitution, reforms that strengthened regulatory transparency, reorganized public institutions, expanded the decentralization process, and introduced new frameworks for renewable energy investment and fiscal incentives. These combined reforms create a clearly identifiable intervention suitable for the SCM. Our contribution to the literature is twofold. First, while SCM has been applied in environmental evaluations in several developing regions, its use remains extremely limited in the MENA context, and to our knowledge, no prior study has applied SCM to assess the environmental implications of Tunisia’s post-2014 institutional transition. Second, by isolating the effect of a multi-dimensional governance and energy reform package on CO₂ emissions, this study moves beyond previous SCM applications that typically focus on single-policy interventions. This allows us to extend the empirical evidence on how institutional quality and reform intensity shape environmental outcomes in politically transitioning economies.

This study makes two main contributions to the literature. First, on the theoretical side, it enriches the debate on the relationship between institutional change and environmental outcomes by examining whether political and economic reforms in a transitioning economy can alter CO₂ emissions trajectories. While prior studies focus primarily on economic growth, energy consumption, or trade dynamics, this study introduces institutional reform as a central explanatory dimension in the environmental, economic nexus within North Africa. Second, from an empirical standpoint, this research provides the first application of the SCM to assess the causal impact of Tunisia’s 2014 reforms on CO₂ emissions. By constructing a data-driven counterfactual for Tunisia using comparable regional economies, the study offers a rigorous empirical assessment that complements and extends previous panel-based or time-series approaches commonly used in the literature.

Against this background, the purpose of this study is to evaluate whether the institutional and economic reforms introduced in Tunisia after 2014 influenced the country’s environmental trajectory, particularly its CO₂ emissions. To guide this analysis, the study addresses the following main research question: Did Tunisia’s post-revolution reforms causally alter the evolution of CO₂ emissions compared to what would have occurred in the absence of these reforms? To answer this question, we employ the SCM to construct a credible counterfactual scenario using comparable North African countries as the donor pool. This approach allows us to isolate the effect of the 2014 reforms and to offer policy-relevant insights into the challenges of aligning institutional transformation with environmental sustainability.

The ensuing sections of this paper are organized as follows: Section 2 provides a review of the relevant literature; Section 3 details the data and presents descriptive statistics; Section 4 outlines the empirical methodology employed; Section 5 reports and discusses the findings; and finally, Section 6 concludes and explores the policy implications of this research.

## Literature review

The determinants of CO₂ emissions and their interaction with economic structures, institutional changes, and energy transitions have been widely investigated in the environmental economics literature. To provide a coherent synthesis, this section organizes the existing research into three thematic clusters: (a) studies on the Environmental Kuznets Curve (EKC) and economic growth–environment dynamics, (b) research on energy transition and CO₂ emissions in North Africa, and (c) empirical determinants of CO₂ emissions and the methodological foundations relevant to this study, including justification for the predictors used in SCM.

### EKC framework and economic growth–environment dynamics

A substantial strand of research examines whether economic development affects environmental quality through non-linear patterns, typically assessed using the EKC framework. Rather than elaborating on its theoretical formulation, most studies investigate the empirical validity of the EKC by estimating the relationship between income levels and emissions across different regions and countries [21, 43]. In North Africa, EKC-related evidence remains mixed, with several studies highlighting that the emissions–growth relationship depends heavily on structural and institutional characteristics. For example, Omri et al. [40] and Al-Mulali et al. [9] show that energy intensity, industrial structure, and governance conditions influence whether income growth leads to improvements or deterioration in environmental quality.

Beyond strictly EKC-focused studies, broader research has identified key macro-determinants of CO₂ emissions. Economic activity, population growth, technological change, and consumption patterns consistently emerge as major contributors [16, 48]. For North African economies, factors such as trade openness, industrial diversification, and energy dependency strongly condition CO₂ trajectories [40]. However, as several authors note, post-revolution institutional and economic reforms remain understudied, especially in Tunisia, where governance restructuring and sectoral reforms after 2011 and 2014 may have altered environmental outcomes in ways not yet empirically assessed. This cluster of studies underscores the need to integrate institutional change into environmental analyses, motivating the present research.

### Energy transition and CO₂ dynamics in North Africa

A second major research cluster focuses on the environmental impact of energy consumption, differentiating between non-renewable energy consumption (NREC) and renewable energy consumption (REC). Evidence overwhelmingly shows that dependence on fossil fuels is strongly correlated with increased CO₂ emissions, both globally and in North Africa [9]. Tunisia is no exception: with oil and natural gas constituting the dominant share of its energy mix, CO₂ emissions have risen steadily over recent decades (ANME, 2024). Numerous empirical studies confirm that increased NREC use is associated with deteriorating environmental quality [9].

Conversely, a large body of research finds that expanding renewable energy deployment reduces emissions and enhances sustainable growth. For instance, IPCC [23], IRENA [24], Menegaki [36], and Sadorsky [42] show that REC expansion lowers environmental pressure and improves energy security. In the North African context, Jammazi et al. [25] highlight the potential of renewable technologies to mitigate CO₂ emissions, while ANME (2024) underscores Tunisia’s efforts to adopt solar and wind energy to promote environmental quality. Additional studies demonstrate that the effectiveness of renewable energy in reducing emissions is influenced by factors such as trade diversification [26], financial inclusion [11], productive capacity [39], and environmental policy instruments [20].

Recent studies have also emphasized the complex role of foreign direct investment (FDI), technological advancement, and governance in shaping environmental outcomes. Evidence from newly industrialized countries reveals that both investment flows and technology transfers impact emissions in a context-dependent manner [28]. Similarly, economic policy uncertainty affects energy system efficiency and environmental performance [29]. The role of institutional quality is highlighted by Kilinc-Ata, Kaya, & Barut [30], who find that democratic governance and societal well-being support stronger climate action. Studies focusing on African economies [31, 32] further reveal that even energy-rich countries struggle with sustainable energy access due to structural constraints.

Another important area of inquiry concerns the environmental effects of FDI through the pollution haven or pollution halo mechanisms. Research indicates that the direction and magnitude of the FDI–CO₂ relationship depend on regulatory stringency, sectoral composition, and technological absorption capacity [8, 44, 49]. This heterogeneity reinforces the need for context-specific analyses, particularly in transition economies like Tunisia.

### Empirical determinants, predictor justification, and SCM-relevant literature

A final cluster relates directly to the predictors used in the SCM and the limited use of SCM in North African environmental analyses. The variables included in this study, GDP, FDI, REC, NREC, trade, governance, and welfare, are strongly grounded in empirical research on emerging and transition economies. Economic growth is consistently identified as a major driver of emissions due to its link with industrial expansion and increased energy consumption [9, 21]. Energy composition is also known to significantly influence emissions: fossil fuel dependency raises CO₂ levels, whereas renewable energy adoption mitigates them [28–30, 40]. Institutional quality, trade dynamics, and welfare conditions have likewise been shown to influence environmental performance [30].

In addition, studies examining economic activities and CO₂ emissions in the context of renewable energy adoption [12, 34] highlight the broader structural forces shaping emissions trajectories. However, despite this extensive empirical base, a notable gap persists regarding the specific environmental impacts of Tunisia’s post-2014 institutional and economic reforms. Existing studies rarely assess how governance restructuring, subsidy reforms, or economic liberalization may have altered Tunisia’s emissions path.

Regarding methodology, the use of SCM to construct a credible counterfactual trajectory for Tunisia remains extremely limited in North Africa. Although SCM is widely applied in policy evaluation globally, its environmental applications in the region are virtually non-existent. Recent SCM research provides strong methodological support for the present study. For example, Abadie and Garber [4] strengthen the theoretical foundation of SCM and its inferential properties. Aguilar-Garcia and Ren [7] apply SCM to evaluate climate policy interventions, demonstrating its suitability for environmental governance analysis. Feng et al. [19] employ SCM to assess environmental shocks, illustrating how the method isolates treatment effects in volatile settings. Li et al. [33] apply SCM to energy-sector reforms and highlight its ability to disentangle regulatory impacts from macroeconomic noise. More recently, Du et al. [17] use SCM to evaluate pollution-control policies, reinforcing the method’s value in studying environmental interventions where randomized experiments are impossible.

This growing body of SCM-based environmental applications underscores the relevance of adopting the method in the present study. Thus, the existing literature supports both the chosen predictors and the SCM approach, while also motivating the need for a rigorous causal assessment of Tunisia’s post-2014 reforms.

## Data and descriptive statistics

### Data

The primary aim of this study is to evaluate the causal effect of Tunisia’s post-2014 institutional and economic reforms on carbon dioxide (CO₂) emissions, using the SCM approach to construct a credible counterfactual scenario. This approach, as implemented in R using the ‘Synth’ package [2], allows for a rigorous estimation of treatment effects in comparative case studies. To achieve this, we use annual data from the World Bank Development Indicators (WDI, 2025) covering the period 2000 to 2023 for six North African countries: Tunisia, Morocco, Egypt, Libya, Sudan, and Algeria.

The analysis is predicated upon five principal variables, as delineated in Table 1: CO₂ emissions (total, excluding land use, land-use change and forestry - LULUCF), measured as percentage change from 1990 levels; renewable energy consumption (REC), expressed as a percentage of total final energy consumption (% of TFEC); gross domestic product (GDP), measured in constant 2015 US dollars (USD); non-renewable energy consumption (NREC), expressed as a percentage of total energy use; and foreign direct investment (FDI), net inflows, measured as a percentage of GDP (% of GDP). This comprehensive dataset enables a robust comparative analysis of Tunisia’s environmental trajectory relative to a synthetic control group composed of its regional peers. Data selection was predicated on maximizing the number of observations, contingent upon their availability within the World Development Indicators (WDI, 2025) database.

**Table 1:** Variables description

Variables	abreviation	unit of measurement	Source
CO_2_ emissions	CO_2_	% change from 1990	WDI (2025)
Gross Domestic Product	GDP	constant 2015 US$	
Renewable energy consumption	REC	% of total final energy consumption	
Non-renewable energy consumption	NREC	% of total energy use	
Foreign direct investment	FDI	% of GDP	

The selection of these predictors follows methodological recommendations from the synthetic control literature, which require variables that capture the structural determinants of the outcome of interest [1]. Economic growth, fossil fuel dependence, renewable energy deployment, and FDI inflows are widely recognized as key drivers of CO₂ emissions in empirical environmental studies. Including these variables ensures that the synthetic control approximates Tunisia’s pre-intervention characteristics in the most relevant economic and energy dimensions. Their selection is therefore grounded both in theoretical considerations and robust empirical evidence, strengthening the credibility of the counterfactual generated by the SCM.

### Descriptive statistics

Descriptive statistics represent a crucial preliminary step before conducting any econometric estimation, as they offer a general overview of the distributional properties and central tendencies of the variables under study. This initial analysis provides valuable insights into the behavior of the data, facilitating a better understanding of potential patterns, heterogeneities, and irregularities that may influence the robustness of subsequent methodological approaches.

As shown in Table 2, CO₂ emissions display a mean of approximately 120.83, with a wide dispersion as indicated by a standard deviation of 72.09, and a positive skewness of 1.05. GDP records a substantial average value of 1.21 × 10^11^, with high variability (standard deviation of 1.03 × 10^11^) and strong positive skewness (1.63), suggesting the presence of extreme values. REC has a mean of 16.97 and a positively skewed distribution (1.65), while NREC shows a higher average of 82.41 but with a negative skewness of −1.69, indicating a concentration of observations near the upper end. FDI averages around 2.27 and exhibits the highest degree of skewness (1.89) and kurtosis (7.48), implying heavy-tailed behavior.

**Table 2:** Descriptive statistics

	CO_2_	GDP	REC	NREC	FDI
Mean	120.8296	1.21*10^11^	16.96935	82.40881	2.266769
Median	104.3872	7.57*10^10^	9.350000	90.54500	1.886191
Maximum	331.7974	4.82*10^11^	80.40000	100.0000	9.424734
Minimum	8.204083	2.80*10^10^	0.100000	17.07000	0.204543
Std. Dev.	72.09176	1.03*10^11^	22.70417	24.71659	1.704363
Skewness	1.052817	1.635043	1.652622	-1.695250	1.894987
Kurtosis	3.771507	5.156950	4.169893	4.190194	7.481513

**Fig. 1:**
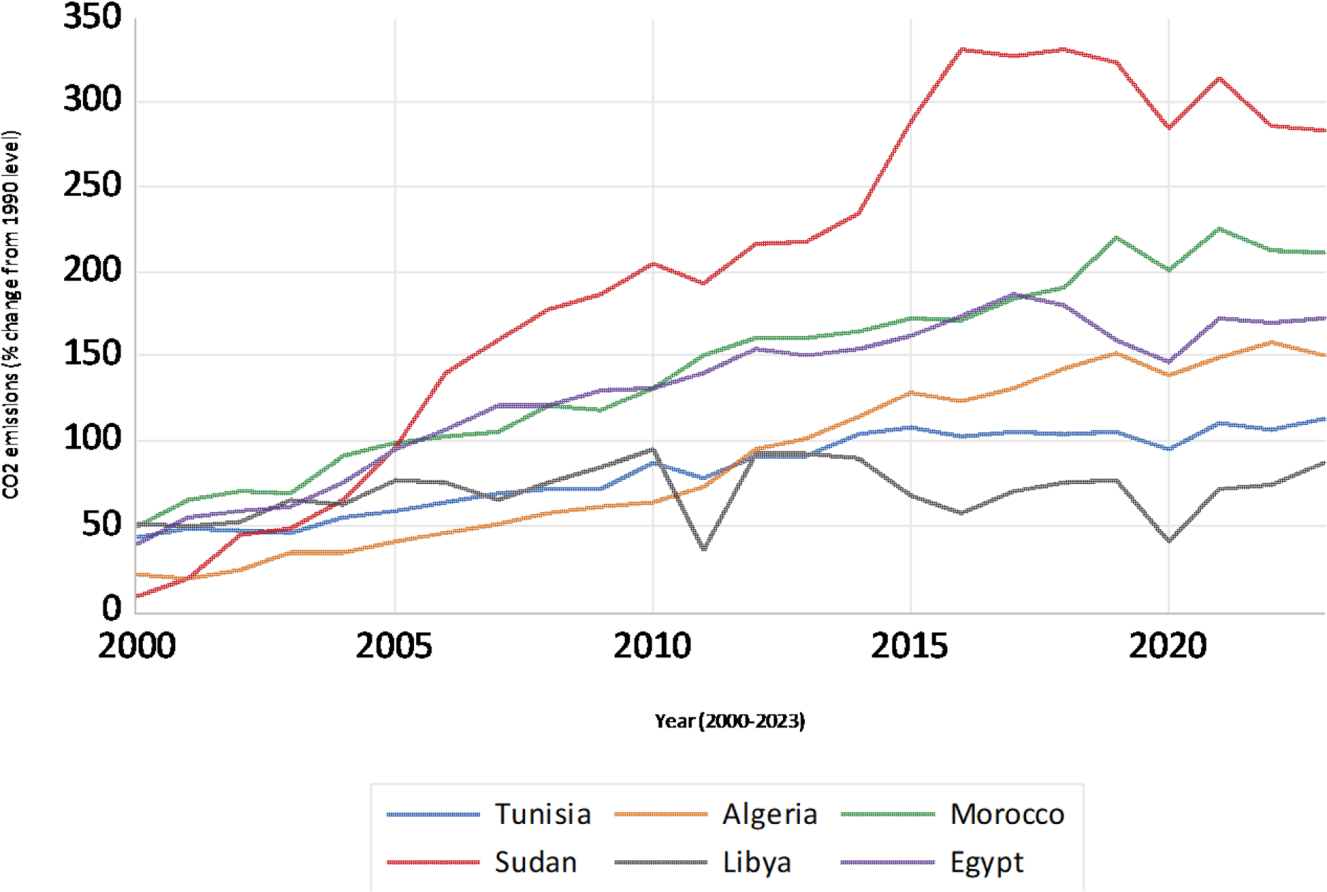
CO₂ emissions in North African countries (% change from 1990 level)

Analysis of CO₂ emissions trends in North African nations from 2000 to 2023, presented in Fig. 1, depicts heterogeneous trajectories relative to 1990 baselines. While a general upward trend is discernible for most countries, the rate and patterns of emissions vary considerably, indicative of differing economic activities and energy policy frameworks. Sudan consistently exhibits the highest levels of CO_2_ emissions relative to 1990, with strong increases until 2017. Conversely, Libya demonstrates a more volatile emissions profile, marked by significant fluctuations. Tunisia shows a relatively steady and moderate rise, Algeria a slow increase, while Morocco and Egypt experience noticeable upward trends that level off in recent years. These divergences underscore the unique environmental characteristics of individual nations within the region and highlight the intricate challenges inherent in mitigating climate change across North Africa.

**Fig. 2:**
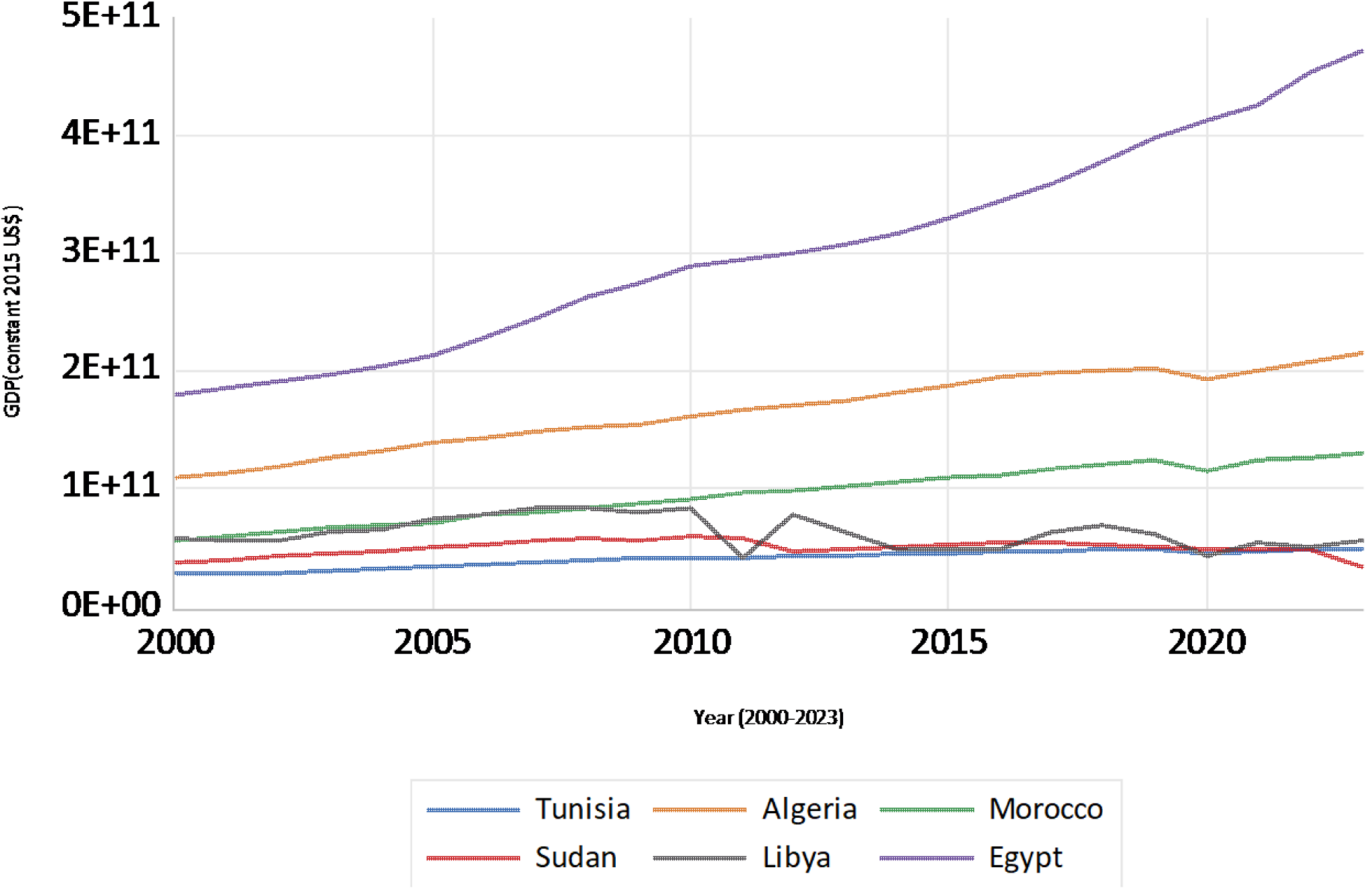
GDP trends in North African countries (constant 2015 US$)

The GDP trajectory across North African countries, presented in Fig. 2, illustrates the heterogeneous landscape of economic development. Egypt demonstrates the most substantial and consistent growth trajectory, significantly outpacing the other nations. Algeria and Morocco exhibit steady, albeit less pronounced, increases in GDP. Conversely, Sudan’s GDP remains relatively flat and stagnant, while Libya experiences notable economic volatility, marked by fluctuations and occasional dips. These diverse patterns highlight the varied economic performance and stability levels within the region, shaped by distinct national policies, resource endowments, and sociopolitical contexts.

**Fig. 3:**
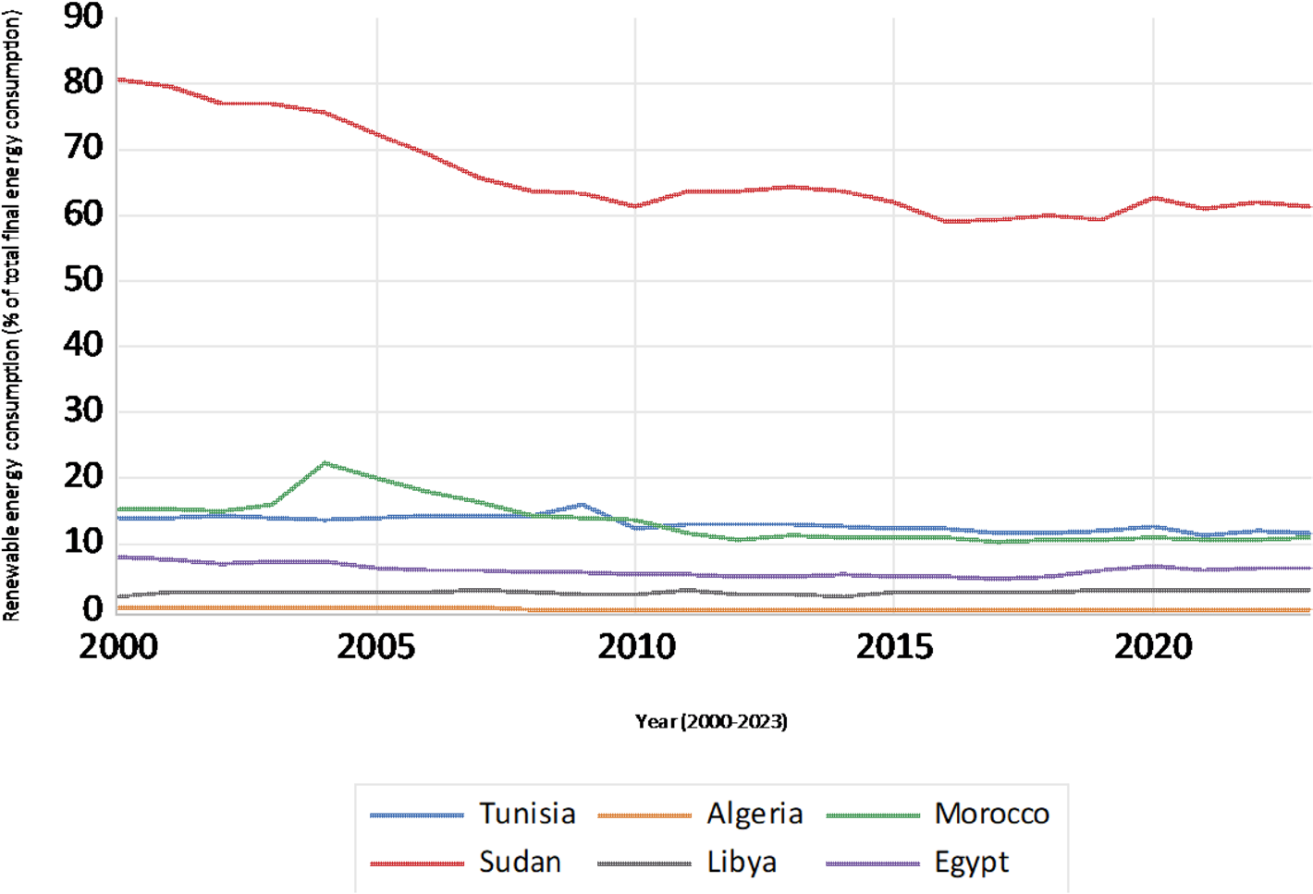
Renewable energy consumption trends (% of total final energy consumption)

The analysis of REC trends for the selected countries, displayed in Fig. 3, demonstrates significant disparities, with Sudan exhibiting the highest levels that gradually decline over time. Conversely, Algeria and Libya demonstrate minimal REC throughout the period. Tunisia, Morocco, and Egypt exhibit relatively low and stable consumption levels, indicating limited adoption of renewable energy sources compared to Sudan. This divergence underscores varying national priorities, investment strategies, and resource endowments that influence the integration of renewable energy within each country’s energy mix.

**Fig. 4:**
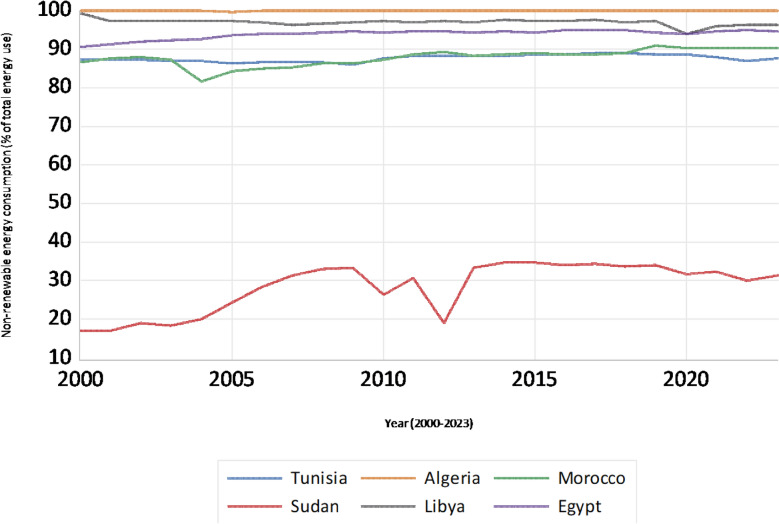
Non-renewable energy consumption trends (% of total energy use)

Figure 4 illustrates the trajectory of NREC for the selected countries, demonstrating a pronounced regional dependence on these energy sources. Except for Sudan, which exhibits a significantly lower and decreasing dependence on non-renewables, most nations maintain relatively high and stable consumption levels, signifying a persistent reliance on fossil fuels and other non-renewable resources to meet their energy demands.

## Methodology

The primary aim of this study is to present the empirical strategy used to evaluate the impact of Tunisia’s institutional reforms, initiated in 2014, on carbon dioxide (CO₂) emissions. Specifically, the analysis examines the role of economic growth (real GDP), renewable energy consumption (REC), non-renewable energy consumption (NREC), and foreign direct investment (FDI) in shaping CO₂ emissions in Tunisia, using other North African countries as the comparison group. Although the present study does not directly estimate an EKC functional form, the theoretical intuition behind the EKC informs the selection of key predictors, particularly GDP and energy consumption variables, given their established relevance in shaping emissions trajectories. Including these predictors in the synthetic control specification ensures that Tunisia is compared to countries with similar economic–environmental dynamics, consistent with prior empirical work grounded in the EKC literature.

As it is impossible to observe both the actual and counterfactual emission paths for the same country, the study applies the SCM, originally developed by Abadie and Gardeazabal [5] and Abadie et al. [1]. This method constructs a counterfactual scenario by creating a weighted combination of control countries, thereby enabling a more credible assessment of the intervention’s causal impact.

The choice of the SCM is motivated by its distinct advantages over alternative causal inference techniques. Unlike the traditional Difference-in-Differences (DiD) approach, which relies on the strong parallel-trends assumption, SCM constructs a transparent and data-driven counterfactual by assigning optimal weights to control units that best reproduce the treated unit’s pre-intervention trajectory [1]. This feature makes SCM particularly suitable when the treated unit exhibits unique economic or environmental dynamics, as is the case for Tunisia. Compared with panel regression methods, SCM minimizes risks of model misspecification and avoids imposing linear functional forms or restrictive parametric assumptions. Furthermore, unlike matching estimators, which match on observed characteristics only, SCM matches on the entire pre-treatment path of the outcome variable, leading to a more credible approximation of the counterfactual. These advantages justify the selection of SCM as the most appropriate method to evaluate the effects of Tunisia’s reforms on CO₂ emissions.

The SCM approach offers a robust framework for evaluating the causal effects of large-scale policy interventions or significant events in cases where traditional comparative case studies may fall short. By synthesizing a control unit that closely mirrors the characteristics of the treated unit (Tunisia) prior to the intervention, the method enables researchers to estimate the intervention’s impact with greater precision and reliability. This methodology is particularly advantageous in the context of macroeconomic and environmental policy analysis, where randomized controlled trials are typically infeasible.

Through the application of SCM, this research aims to generate a synthetic counterpart to Tunisia, thereby providing a credible basis for comparison and allowing for a nuanced examination of the institutional reforms’ effects on CO₂ emissions. This approach not only addresses the counterfactual question of what would have occurred in the absence of the 2014 intervention but also offers valuable insights into the efficacy of Tunisia’s environmental and economic policies in the post-reform era.

Let us consider a setting with $J+1$ units, where unit $j=1$ is the treated unit (Tunisia), and units $j=2,\dots ,J+1$ are the control units (Algeria, Egypt, Libya, Morocco, Sudan). Let ${T}_{0}$​ denote the number of pre-intervention periods and $T$ the total number of periods.

The observed outcome for unit $i$ at time $t$ is defined as:

1 $$ Y_{it} = Y_{it}^{N} + \alpha_{it} D_{it} ,\;{\mathrm{for}}\;i = 1, \ldots ,J + 1\;{\mathrm{and}}\;t = 1, \ldots ,T $$

where ${Y}_{it}$ is the observed outcome (e.g., log of CO₂ emissions); ${Y}_{it}^{N}$ defines the outcome that would be observed in the absence of treatment; ${D}_{it}$ is the indicator variable equal to $1$ if unit $i$ is treated at time ttt, and 0 otherwise; ${\alpha }_{it}$ the treatment effect for unit $i$ at time $t$.

The primary goal is to estimate ​ ${\alpha }_{1t}$ for $t>{T}_{0}$, which represents the causal effect of the intervention on Tunisia’s CO₂ emissions. The synthetic control is created by selecting a set of weights $W=\left({w}_{2},\dots ,{w}_{J+1}\right)$, where ${w}_{j}\ge 0$ and $\sum_{j=2}^{J+1}{w}_{j}=1$ such that the weighted average of the control units closely approximates the pre-treatment characteristics of the treated unit.

Formally, the SCM minimizes the distance between the treated unit and its synthetic counterpart over a vector of predictor variables $X$ by solving the following optimization problem:

2 $$\underset{W}{\mathrm{min}}{\left({X}_{1}-{X}_{0}W\right)}{\prime}V\left({X}_{1}-{X}_{0}W\right)$$

where ${X}_{1}$ denotes the vector of pre-treatment characteristics for the treated unit; ${X}_{0}$ matrix of the same variables for the control units; $V$ indicates a symmetric and positive semi-definite matrix that assigns importance to each predictor.

Once the optimal weights are identified, the synthetic control outcome is computed as:

3 $$Y_{1t}^{SC} = \sum\nolimits_{j = 2}^{J + 1} {w_{j}^{*} Y_{jt} \;{\;\;{for}}\;t = 1, \ldots T} $$

The treatment effect is then given by:

4 $$\hat{\alpha }_{1t} = Y_{1t} - Y_{1t}^{SC} \;{\;\;{for}}\;t\,{ > }\,T_{0} $$

This study examines Tunisia’s institutional changes implemented in 2014, focusing on their environmental impact. We selected Tunisia due to its unique position as a North African country undergoing significant democratic and economic reforms post-2011 [14], with 2014 marking a pivotal year of constitutional change and structural reforms [46]. The analysis compares Tunisia to a group of similar countries: Algeria, Egypt, Libya, Morocco, and Sudan. Our primary interest is CO₂ emissions, measured using logarithms to capture relative changes and facilitate cross-country comparisons, following the approach of Abadie et al. [3]. We also consider other influential factors, including GDP, REC, NREC, and FDI, all expressed in logarithmic form to address potential non-linearities and improve interpretability (Wooldridge, 2016). The logarithmic transformation allows us to examine percentage changes rather than absolute differences, providing a more nuanced understanding of the relationships between economic indicators and environmental outcomes [13]. By employing the SCM developed by Abadie et al. [1] and Abadie and Gardeazabal [5], we construct a hypothetical version of Tunisia that didn’t experience the 2014 changes, enabling us to estimate the counterfactual CO₂ emissions trajectory and quantify the impact of the reforms. This approach offers valuable insights into how Tunisia’s political and economic transitions have affected its environmental performance, particularly regarding GHG emissions, in the context of its unique reform process [40].

The SCM follows a structured procedure designed to estimate the causal impact of an intervention. First, the synthetic weights are estimated by assigning a set of optimal weights to the control units in the donor pool. These weights are chosen so that the weighted average of the control units closely approximates the pre-intervention characteristics of the treated unit.

The donor pool is intentionally restricted to North African countries, Algeria, Egypt, Libya, Morocco, and Sudan, to ensure that the synthetic unit is constructed from countries sharing the same geographical region, structural characteristics, institutional trajectories, and exposure to similar political and economic shocks. Maintaining regional homogeneity is important in SCM applications because it reduces unobserved heterogeneity and enhances the credibility of the counterfactual. Although this restriction may produce weight concentration on structurally similar units such as Libya, the regional focus avoids inappropriate comparisons with countries outside the North African context and preserves the validity of the identification strategy.

Second, the treatment effect is inferred by comparing the post-intervention outcome trajectory of the treated unit to that of its synthetic counterpart, with graphical tools such as trajectory plots and gap plots aiding in visual interpretation. Finally, to validate the estimated effect, placebo tests were conducted.

## Results and discussion

It is important to note that the SCM identifies relative divergence in outcomes over time and does not allow for causal attribution to individual policy measures implemented during the transition period. This study applies the SCM to evaluate the impact of Tunisia’s post-2014 reforms on CO₂ emissions. The SCM is particularly well-suited for this analysis, as it constructs a counterfactual scenario by creating a “synthetic” version of Tunisia derived from a weighted combination of comparable countries. This approach provides a more accurate estimate of policy impacts than alternative methods, such as Difference-in-Differences (DiD), which rely on the assumption of parallel pre-treatment trends. By ensuring that the synthetic Tunisia closely mirrors the real country’s pre-2014 economic and emissions patterns, the SCM enables a more robust assessment of how policy interventions influence outcomes over time.

Statistically, the W-weights reflect the importance of the control countries to re-create the synthetic control. A weight close to one indicates how well the control country replicates all the conditions of the treated unit. In this case, the SCM^1^ constructs a counterfactual Tunisia by assigning weights to a pool of potential control countries (Algeria, Egypt, Libya, Morocco, and Sudan).

These weights, presented in Table 3, reflect the relative contribution of each country in replicating Tunisia’s pre-2014 characteristics. The results indicate that Libya (80%)^2^ is by far the dominant contributor to synthetic Tunisia. In contrast, Morocco (10%) and Sudan (10%) each contribute a small portion. Both Algeria and Egypt receive weights of 0. This suggests that their economic and energy sector trajectories before 2014 do not align well with those of Tunisia compared to Libya, Morocco, and Sudan; thus, these countries are assigned a zero weight in the synthetic control. The restricted regional donor pool of five countries results in this substantial Libya weighting (80%), reflecting a structural limitation inherent to SCM applications in North Africa [1]. This concentration, while constraining inferential precision, is characteristic of small-sample regional designs. Importantly, leave-one-out robustness checks (Table 8) demonstrate that the post-2014 emissions divergence pattern remains stable across alternative specifications.

**Table 3:** Plausible country weights in synthetic Tunisia

Country	Weight
Algeria	0.00
Egypt	0.00
Libya	0.80
Morocco	0.1
Sudan	0.1

The substantial weight of Libya in the composition of synthetic Tunisia likely reflects its pre-2014 dependence on oil exports and its comparable challenges in achieving economic diversification. However, the model’s validity is undermined by the inclusion of variables and characteristics from other countries that are not well-suited to this methodology and that may have limited or no relevance to CO₂ emission dynamics. In contrast, Morocco’s relatively small weight may be attributed to its early adoption of renewable energy initiatives [24], which differentiates its emissions profile from Tunisia’s. Similarly, the exclusion of Algeria and Egypt could stem from their more diversified economic structures and distinct policy agendas [18]. These patterns underscore the complex interaction between economic structure, policy orientation, and the constraints of data-driven modeling in shaping the synthetic control outcome.

**Fig. 5:**
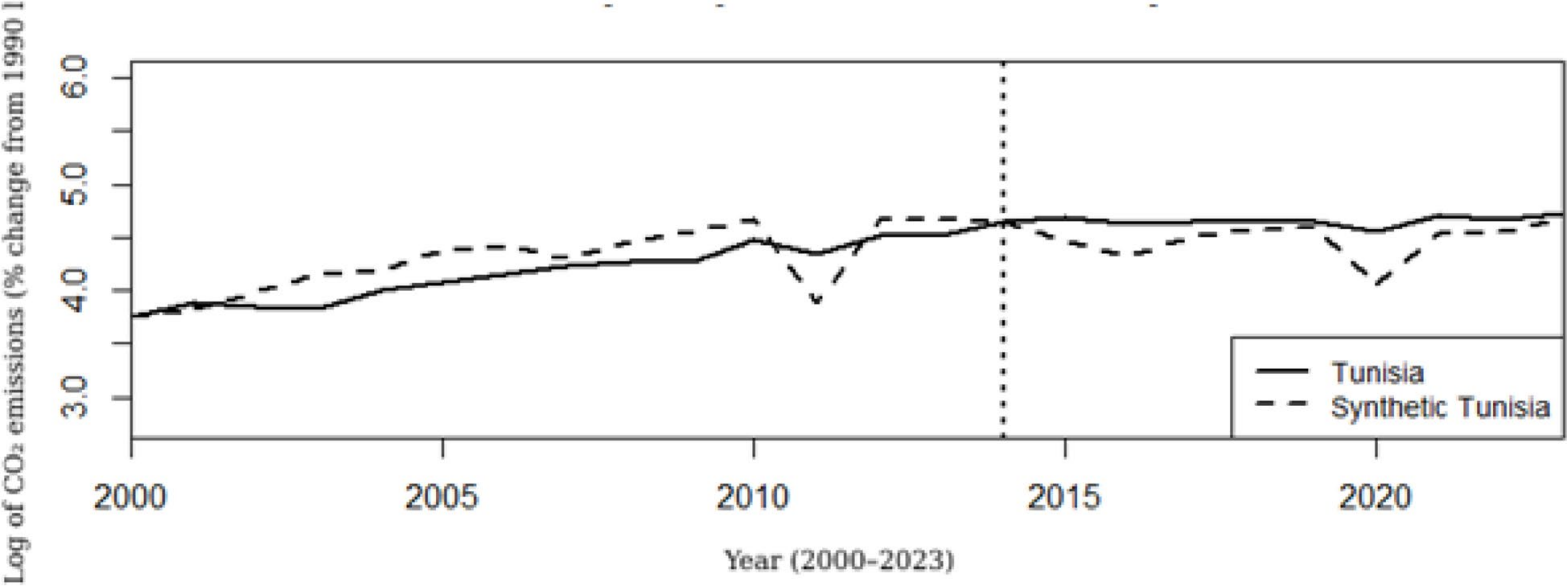
CO₂ emissions trajectory: Tunisia versus synthetic control

Figure 5 illustrates the evolution of CO₂ emissions in Tunisia between 2000 and 2024, compared to a synthetic version of Tunisia constructed from a group of structurally similar countries. The solid line depicts actual CO₂ emissions in Tunisia (in logarithmic scale), while the dashed line represents the emissions trajectory of Synthetic Tunisia, which closely tracks Tunisia’s observed path prior to 2014. This close pre-2014 alignment indicates that the synthetic control provides a credible approximation of Tunisia’s counterfactual emissions trajectory in the absence of major institutional changes. A noticeable drop in emissions around 2010 may be associated with the Arab Spring, which disrupted economic activity across the country.

Following the post-2014 institutional transition, the two trajectories begin to diverge. Tunisia’s observed CO₂ emissions continue to increase, particularly between 2014 and 2018, before entering a phase of relative stabilization, whereas emissions in the synthetic control decline over the same period. This widening gap reflects a divergence in emissions trajectories during the post-2014 transition rather than a direct causal effect of specific policy interventions. The observed pattern suggests that the institutional and economic reforms implemented after 2014 coincided with a less favorable emissions trajectory relative to the synthetic counterfactual.

While this divergence raises important questions regarding the environmental orientation of the post-2014 transition, it should not be interpreted as definitive evidence of reform ineffectiveness. Instead, it may reflect broader structural factors, such as continued reliance on fossil fuels, delays in renewable energy deployment, or investment priorities not fully aligned with environmental objectives, that shaped emissions dynamics during this period.

The post-2014 divergence between Tunisia’s observed CO₂ emissions and its synthetic counterfactual suggests that the institutional transition period coincided with a less favorable emissions trajectory rather than an improvement in environmental performance. Although the Renewable Energy Law (Law No. 2015-12) marked a formal commitment to clean energy development, delays in the adoption of implementing decrees and limitations in grid access significantly constrained large-scale renewable energy deployment during the post-2014 period. At the same time, the persistence of fossil-fuel subsidies reduced price incentives for energy transition, thereby sustaining reliance on carbon-intensive energy sources and contributing to continued emissions growth. These challenges were compounded by fragmented institutional coordination and limited regulatory enforcement capacity, which weakened the translation of reform commitments into effective environmental outcomes.

The synthetic control analysis results indicate a distinct divergence in CO₂ emissions trajectories between observed post-2014 outcomes in Tunisia and the constructed counterfactual scenario, implicating the institutional and economic reforms as influential factors shaping environmental performance. One credible explanation centers on the structural transformation of the Tunisian economy during the reform period, characterized by a deceleration in industrial output and resultant shifts in energy consumption patterns. This interpretation is consistent with recent empirical work demonstrating that institutional transitions or economic restructuring phases frequently induce short-term environmental volatility, driven by alterations in investment flows, productive capacity, and energy demand [28–30, 40]. Furthermore, the growing discrepancy between actual and synthetic emissions after the reforms may be attributable to lagged renewable energy adoption, ongoing reliance on fossil fuels, and prevailing policy uncertainty, variables extensively recognized as impediments to environmental progress in developing and reforming contexts. These observations align with findings from OECD and emerging economies, emphasizing the critical roles of governance quality, rule of law, and policy consistency in enabling effective emissions mitigation [30]. Collectively, our findings suggest that although Tunisia’s post-revolution reforms aimed at institutional modernization and economic revitalization, they have yet to yield immediate environmental benefits. This underscores the imperative for complementary measures such as sustained green investments, regulatory clarity, and diversification of energy sources to fully leverage institutional reforms for environmental sustainability.

Table 4 displays the pre-intervention values of the key predictors for Tunisia (the treated unit), its synthetic counterpart, and the average of the control group. Constructed to closely match Tunisia’s pre-intervention characteristics through a weighted combination of control units, the synthetic version demonstrates a reasonable approximation of Tunisia’s log of GDP (24.922 vs. 24.303) and NREC (4.426 vs. 4.467). However, notable discrepancies exist, particularly in REC and FDI, where Tunisia exhibits a higher reliance on REC (2.640) and greater inflows of FDI (1.060) compared to its synthetic counterpart (1.497 and 0.535, respectively) and the control group averages. These differences suggest that while the constructed synthetic control performs adequately for certain economic and energy indicators, it tends to underrepresent Tunisia’s renewable energy profile and investment levels, pointing to potential areas for model refinement.

**Table 4:** Predictors’ values of Tunisia and Synthetic Tunisia

Predictor	Tunisia (Treated)	Synthetic Tunisia	Sample Mean (Control Group)
lnGDP	24.303	24.922	25.303
lnREC	2.640	1.497	1.739
lnNREC	4.467	4.426	4.273
lnFDI	1.060	0.535	0.646

In the context of SCM analyses, placebo tests serve as a critical robustness check, designed to evaluate the statistical significance of the estimated treatment effect. Given that the SCM aims to isolate the causal impact of an intervention, it is essential to assess whether the observed effect is truly attributable to the intervention or simply a product of chance or idiosyncratic factors. To this end, placebo tests involve the application of the SCM to control units, treating them as if they had undergone the intervention, thereby generating placebo treatment effects. By comparing the magnitude of the actual treatment effect on the treated unit with the distribution of these placebo effects, researchers can assess the likelihood that the observed effect is a genuine consequence of the intervention, rather than a spurious correlation.

To interpret the observed divergence between Tunisia and its synthetic counterfactual after 2014, it is essential to consider the broader institutional and governance context in which these reforms were implemented. Despite the ambitious reform initiatives introduced after 2014, Tunisia’s environmental outcomes cannot be understood solely through the adoption of new policies; rather, they depend heavily on the underlying institutional capacity to implement, coordinate, and enforce them [27, 35]. Research in environmental governance highlights that successful climate and energy transitions require strong regulatory institutions, coherent policy frameworks, and effective state capacity, elements that remain fragile in many developing and post-transition economies. Weak enforcement mechanisms, fragmented institutional responsibilities, and limited administrative resources can weaken the impact of environmental reforms, even when policies are well-designed on paper. In Tunisia, challenges such as overlapping mandates across ministries, inconsistent regulatory enforcement, and budgetary constraints have been shown to hinder the effectiveness of renewable energy promotion, subsidy restructuring, and environmental monitoring [47]. This perspective helps explain why reforms may not have translated into measurable reductions in CO₂ emissions, underscoring the importance of institutional coherence and governance quality when evaluating policy outcomes.

Table 5 displays the weights derived from placebo tests, where each control unit is iteratively treated as the intervention recipient to assess pre-treatment similarity. The results indicate that a limited subset of characteristics from the actual treated unit is adequately replicated across the control pool. Notably, in many placebo tests, a single control unit, frequently Libya, accounts for a disproportionately large share of the synthetic control, suggesting that reliance only on those variables.

**Table 5:** Placebo test results: country weights

Treated Unit	Algeria	Morocco	Sudan	Libya	Egypt
Tunisia	0.046	0.954	0.00	0.00	0.00
Algeria	0.227	0.000	0.773	0.00	0.00
Morocco	0.283	0.000	0.000	0.000	0.338
Sudan	0.000	0.559	0.000	0.154	0.287
Libya	0.000	0.000	1.000	0.00	0.00
Egypt	0.000	0.800	0.100	0.100	0.00

As reported in Table 6, the predictor balance results compare Tunisia with its synthetic counterpart based on key explanatory variables before the intervention period. The values of X_1_​ represent Tunisia, while X_0_​ refers to the synthetic control. The difference between these values (Diff) and the associated predictor weights (V_Weight) provides insights into the quality of the matching process. Notably, the variable lnNREC exhibits a minimal difference (0.029), indicating a close alignment, and it receives a considerable weight (0.247), reflecting its importance in constructing the synthetic control. In contrast, lnREC presents the largest imbalance (−2.157), yet it is assigned the highest weight (0.336), suggesting that despite the gap, the optimization algorithm placed significant emphasis on this predictor. The other variables, such as lnFDI and, show moderate discrepancies (−0.286 and 0.683, respectively), with corresponding weights of 0.281 and 0.136. These results imply that the synthetic control was calibrated by prioritizing the most influential predictors, especially lnREC and lnNREC, in order to replicate Tunisia’s pre-treatment characteristics as accurately as possible.

**Table 6:** Predictor balance: Tunisia and control units

Unit	Predictor	X_1_	X_0_	Diff	V_Weight
Tunisia	lnGDP	25,6806824	24,99753426	0,68314814	0,136138104
Tunisia	lnREC	-1,115139498	1,041873295	-2,157012793	0,335830818
Tunisia	lnNREC	4,603053376	4,573753888	0,029299488	0,246961781
Tunisia	lnFDI	0,118445263	0,404022005	-0,285576742	0,281069297
Algeria	lnGDP	26,18921711	25,21356798	0,975649128	0,001049693
Algeria	lnREC	1,849973061	1,846388162	0,003584899	0,912391602
Algeria	lnNREC	4,534900854	4,491876594	0,04302426	0,084606298
Algeria	lnFDI	0,545912108	0,657918519	-0,112006411	0,001952407
Morocco	lnGDP	24,93967258	25,35771143	-0,418038855	0,000787187
Morocco	lnREC	1,002636238	0,999933791	0,002702447	0,864386327
Morocco	lnNREC	4,575640402	4,537475963	0,038164438	0,133094126
Morocco	lnFDI	0,397132561	0,559010905	-0,161878345	0,001732359
Sudan	lnGDP	25,07628576	25,40761065	-0,331324895	5,16152E-08
Sudan	lnREC	2,71700363	2,444121041	0,272882589	0,049418826
Sudan	lnNREC	4,459374789	4,308766584	0,150608205	0,007530534
Sudan	lnFDI	0,816472599	0,817719927	-0,001247328	0,943050588
Libya	lnGDP	24,62680589	25,07628573	-0,449479836	4,71117E-05
Libya	lnREC	4,239585703	2,717003443	1,52258226	0,510569002
Libya	lnNREC	3,19351751	4,4593748	-1,26585729	0,487056822

X_1_ denotes the average value of the predictor for the treated country (2000–2013); X_0_ indicates the synthetic counterpart’s average value for the same period; Diff is the difference = X_1_−X_0_ (how well the synthetic unit approximates the treated one); and V_Weight is the Weight assigned to each predictor during optimization.

Table 7 shows the Root Mean Square Prediction Error (RMSPE) ratios for Tunisia and the control countries, comparing the model’s accuracy before and after the intervention. Tunisia has an RMSPE ratio of 1.19, meaning the prediction error increased slightly after the intervention, which may indicate an effect of the policy or event. Most control countries have RMSPE ratios close to or less than 1, such as Egypt (0.93), Algeria (0.92), and Sudan (1.00), showing that their prediction errors remained stable over time. Morocco has the highest ratio (1.99), suggesting a large increase in prediction error, while Libya has the lowest (0.66), indicating a better fit after the intervention. These findings suggest that Tunisia experienced a change in its outcome that is not seen in most of the control countries, which supports the reliability of the estimated treatment effect.

**Table 7:** RMSPE ratios for Tunisia and control units

Unit	RMSPE_Pre	RMSPE_Post	RMSPE_Ratio
Tunisia	0,57443193	0,680771619	1,18512148
Algeria	0,150809236	0,139477327	0,924859316
Morocco	0,341336279	0,677630324	1,985227956
Sudan	0,222340289	0,223156659	1,003671718
Libya	0,675485057	0,448691206	0,664250381
Egypt	0,230218691	0,21403834	0,929717477

To ensure that the findings reported in Table 7 are not driven by predictor imbalance, particularly the discrepancies observed in lnREC and lnFDI, we conducted a series of robustness checks by re-estimating the SCM under alternative predictor specifications. Three specifications were tested: (i) the baseline model using all predictors included in Table 7, (ii) a specification excluding lnREC, and (iii) a specification excluding lnFDI, as these two variables showed the largest pre-treatment mismatch and highest V-weights.

Table 8 summarises the pre- and post-treatment RMSPE, the RMSPE ratios, and the average post-2014 treatment effect (ATT) obtained from each specification. The robustness results reinforce the validity of the main findings. In the standard model, the pre-treatment RMSPE is 0.448, while the post-treatment RMSPE is 0.588, yielding a ratio of 1.31 and an average post-2014 ATT of –0.583. This indicates that Tunisia’s CO₂ emissions remained higher than those of its synthetic counterpart following the 2014 reforms. When lnREC is excluded, the results remain highly consistent: the ATT becomes –0.694 and the RMSPE ratio increases only slightly to 1.41. This demonstrates that renewable energy consumption, despite its imbalance, is not disproportionately influencing the outcome. When lnFDI is removed, the model achieves an even better pre-intervention fit (RMSPE ratio = 0.68), and although the post-treatment ATT decreases in magnitude (–0.187), its sign remains negative, confirming that the treatment effect is qualitatively unchanged. Importantly, across all three specifications, the synthetic control continues to diverge downward relative to Tunisia after 2014, indicating that the observed post-reform increase in emissions is not attributable to a single predictor.

**Table 8:** Robustness analysis of synthetic control estimates under alternative predictor specifications

specification	Predictors	rmspe_pre	rmspe_post	rmspe_ratio	ATT_post_mean
standard	lnGDP,lnREC,lnNREC,lnFDI	0.447648	0.588424	1.314478	-0.583302
no_lnREC	lnGDP,lnNREC,lnFDI	0.495489	0.700838	1.414436	-0.694242
no_lnFDI	lnGDP,lnREC,lnNREC,lnFDI	0.299327	0.203719	0.680592	-0.187467

Overall, the consistency of the treatment effect across all alternative specifications confirms that the main SCM results are robust. The observed post-2014 divergence between Tunisia and its synthetic control is therefore not an artefact of predictor imbalance but reflects a stable and reproducible pattern across multiple model configurations.

Notably, REC and FDI—both theoretically central to environmental interpretation—exhibit poor pre-treatment matching (lnREC: Diff = -2.157; lnFDI: Diff = -0.286) alongside high V-weights (0.336/0.281, Table 6). This pattern suggests the synthetic control is primarily driven by macro-structural similarity (GDP/NREC) rather than environmental-energy composition, warranting cautious interpretation of sector-specific environmental inferences. Importantly, leave-one-out robustness checks excluding these predictors individually (Table 8) confirm the post-2014 divergence pattern remains stable across specifications [1].

**Fig. 6:**
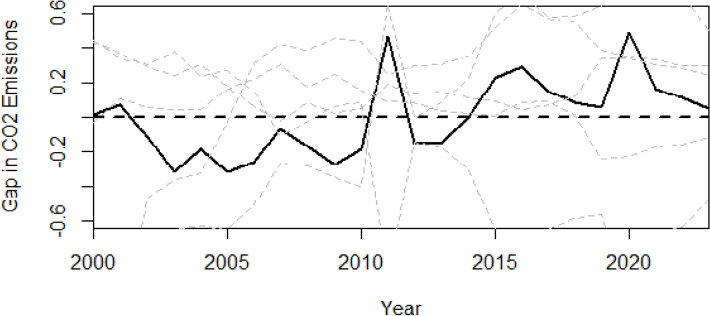
Placebo test: Tunisia vs Controls

Figure 6 presents the results of the placebo tests, which assess whether the observed changes in Tunisia’s CO₂ emissions can be plausibly attributed to the implemented policies. The graph depicts the gap between actual and synthetic CO₂ emissions over time, with the black line representing Tunisia and the grey lines representing placebo estimates for control countries, assuming they experienced the same policy intervention. Ideally, the black line would diverge significantly from the grey lines following the policy implementation in 2014. Although the divergence observed between Tunisia and its synthetic control after 2014 suggests an increase in emissions relative to the counterfactual scenario, this trend should not be construed as conclusive causal evidence that the institutional and economic reforms directly caused a rise in CO₂ emissions. The placebo tests reveal only moderate differentiation between Tunisia and the donor countries, indicating that caution is warranted when attributing the post-2014 emissions trajectory solely to the implemented policy reforms. Multiple alternative factors may explain the observed increase. First, the post-2011 Arab Spring period was characterized by a gradual economic recovery, likely driving higher energy demand and industrial activity independently of the 2014 reforms. Second, fluctuations in international energy prices, especially rising fossil fuel costs, may have influenced consumption patterns, contributing to elevated emissions. Third, changes in foreign direct investment and external financing conditions could have affected the economic structure and energy intensity during this timeframe. Collectively, these considerations suggest that the post-2014 emissions divergence results from an interplay between domestic reforms and broader economic and external influences, rather than a direct causal impact of institutional changes alone.

## Conclusion and policy recommendations

This study provides a rigorous empirical assessment of the evolution of Tunisia’s CO₂ emissions trajectory during the post-2014 institutional and economic transition using the SCM approach. Rather than identifying a direct causal impact of specific reforms, our findings indicate that, despite intentions to foster sustainable economic growth, the post-2014 transition did not coincide with statistically significant or immediate reductions in emissions relative to the synthetic counterfactual. However, placebo tests and RMSPE ratios indicate this divergence is only weakly distinguishable from donor-country placebos, warranting cautious interpretation of the findings. The absence of a clear and persistent divergence between Tunisia’s observed emissions trajectory and that of the synthetic control highlights the difficulty of achieving environmental improvements during periods of deep structural economic and policy transformation and suggests that the estimated treatment effect remains weak or indistinguishable within the study period.

Policy recommendations from this analysis emphasize the need to move beyond generic sustainability commitments toward reform-specific implementation strategies. In particular, greater priority should be given to the full operationalisation of the Renewable Energy Law (Law No. 2015-12), including the timely implementation of decrees, improved grid access, and investment facilitation mechanisms. A gradual and socially balanced phase-out of fossil-fuel subsidies is also essential to realign price incentives in favor of cleaner energy sources and accelerate energy diversification. Strengthening governance quality, enhancing inter-ministerial coordination, ensuring policy continuity, and reducing economic policy uncertainty remain vital to unlocking the environmental benefits of institutional reforms. Furthermore, explicitly embedding environmental objectives within economic, industrial, and energy policy design is crucial to ensure that growth strategies are consistent with long-term sustainability goals rather than short-term macroeconomic stabilization priorities.

Several limitations should be considered. The validity of the SCM depends on the availability and comparability of suitable control countries, and the relatively small donor pool and weight concentration limit the precision with which Tunisia’s emissions trajectory can be distinguished from placebo cases. Data constraints also limited the inclusion of more granular sectoral, technological, or policy variables that could influence emissions. Additionally, as SCM captures relative outcome divergence rather than isolated policy effects, unobserved confounders or dynamic spillover effects may influence the results, necessitating cautious interpretation.

Future research should address these limitations by incorporating higher-resolution datasets, extending the analysis time horizon, and exploring complementary methodologies to capture complex environmental–economic dynamics more fully. Comparative studies examining similar political and economic transitions in other MENA or emerging economies would further enhance the generalizability and policy relevance of findings. Investigations into specific policy instruments, renewable energy financing mechanisms, technological innovation pathways, and social dimensions of climate policy implementation also represent promising avenues for advancing understanding of sustainable development transitions.

Overall, these findings should be interpreted as context-specific evidence of divergence in Tunisia’s emissions trajectory during the post-2014 institutional transition, rather than definitive proof of the causal effectiveness or ineffectiveness of individual reforms.

## Data Availability

No datasets were generated or analysed during the current study.

## Footnotes

1 All variables used in the Synthetic Control Method analysis were transformed into their logarithmic forms before model estimation. This transformation helps stabilize variance, reduce skewness, and improve the comparability between Tunisia and donor countries, thereby enhancing the reliability of the synthetic control construction.

2 To examine whether Libya’s dominant weight affects the results, we conducted a sensitivity test excluding Libya from the donor pool. Although this altered the synthetic trajectory slightly due to Tunisia’s similarity with Libya, the post-2014 divergence with the synthetic control remained. This confirms the treatment effect is robust and not solely driven by Libya’s inclusion.
